# Differential Expression of Apoptosis-Stimulating Proteins of p53 (ASPPs) Between Langerhans Cell Histiocytosis and Langerhans Cell Sarcoma

**DOI:** 10.3390/diagnostics16101418

**Published:** 2026-05-07

**Authors:** Changsong Wang, Naijun Fan, Tian Yun, Fulin Li, Nianlong Meng, Yaxi Wang, Junling An, Xuexia Lyu

**Affiliations:** 1 Department of Pathology, The 989th Hospital of the PLA Joint Logistic Support Force, Luoyang 471031, China; wangtmmu150@163.com (C.W.);; 2 Department of Pathology and Pathophysiology, School of Basic Medicine, Henan University of Science and Technology, Luoyang 471031, China

**Keywords:** Langerhans cell sarcoma, Langerhans cell histiocytosis, MAP2K1, ASPPs, molecular marker

## Abstract

**Objectives**: Langerhans cell sarcoma (LCS) is a very rare, highly malignant tumor that originates from Langerhans cells. The differential diagnosis of LCS and Langerhans cell histiocytosis (LCH) still faces limitations, and the molecular changes involved in LCS are unclear. Molecular biomarkers and immunophenotypes may help distinguish between LCS and LCH. In this manuscript, the pathological and molecular markers in LCS are explored. **Methods**: The expression patterns of ASPP1, ASPP2, and inhibitor of apoptosis-stimulating p53 protein (iASPP) were examined using the immunohistochemical method and immunofluorescence staining. Then, genetic features, such as B-Raf proto-oncogene, serine/threonine kinase (BRAF) V600E, K-ras, and ROS proto-oncogene 1, receptor tyrosine kinase (ROS1), were assayed using the amplification refractory mutation system (ARMS) method. Finally, whole-exon sequencing of LCS was performed. **Results**: Immunohistochemically, in all samples of LCS, ASPP2 was detected in ovoid and elliptic tumor cells. In the case of LCH, ASPP2 was expressed not only in ovoid and elliptic cells but also in histiocytic cells. The expression of iASPP was observed in five cases LCS (5/6), and no positive reaction was observed in the case of LCH. No ASPP1 expression was observed in LCH and LCS. During triple-color immunofluorescence analysis, ASPP2 and iASPP were co-expressed on Langerin^+^ LCS tumor cells. No mutations of BRAF V600E, K-ras, or ROS1 were detected in LCH and LCS. No gene mutation or rearrangement was detected in LCS except for the MAP2K1 gene. The mutation site was nonsynonymous in 607 bp of MAP2K1, resulting in a change from base G to A; thus, the amino acid E changed to K at the 203 site (4/6, 66.67%). **Conclusions**: Combined detection of ASPP2 and iASPP in tissue samples may provide valuable markers to differentiate between LCH and LCS. The MAP2K1 variants c.607G > A is the first potential marker to be reported in LCS.

## 1. Introduction

According to the 2016 World Health Organization (WHO) classification of lymphohemopoietic tumors, Langerhans cell (LC) neoplasms are divided into Langerhans cell histiocytosis (LCH) and Langerhans cell sarcoma (LCS). At present, the combination of histopathological examination and immunohistochemical analysis is routinely used for diagnosis of Langerhans cell neoplasms. Furthermore, when Langerhans cell neoplasms are diagnosed, further differentiation is necessary to determine a diagnosis of LCH or LCS. However, in pathological practice, the differential diagnosis of LCS and LCH is challenging, as both types share the same cell origin, have similar histological features, immunophenotypes, and ultra-structure characteristics. Hence, other parameters are needed to establish the differentiation between LCH and LCS, as well as molecular markers and mechanisms related to LCS. See the end of the documents for further details on references.

Apoptosis-stimulating proteins of p53 (ASPPs) refer to a family of proteins that regulate wild-type p53 and function as tumor suppressors, comprising ASPP1, ASPP2, and iASPP. ASPP1 and ASPP2 promote cell apoptosis, while overexpression of iASPP inhibits apoptotic cell death after DNA damage [1]. In multiple human tumor tissues and cancer cells, the aberrant expressions of ASPP1, ASPP2, and iASPP have been observed. In particular, lowly expressed ASPP2 and highly expressed iASPP in cancers are associated with worse disease status, therapy resistance, and poor survival in patients with malignant tumors [2,3,4]. As an important tumor-associated gene, does ASPPs play a role in LC neoplasms? If it does, through what pathway or mechanism does it exert its effect? In this paper, we not only examine the expression patterns of ASPP1, ASPP2, and iASPP in LC neoplasms but also their potential for differentiating between LCS and LCH. At the same time, the molecular and genetic characteristics of LCS are explored to uncover the mechanisms behind this type of disease.

## 2. Materials and Methods

### 2.1. Patients and Tissue Samples

In this study, 6 samples of LCS and 6 samples of LCH were collected from the Department of Pathology, the 989th Hospital of the PLA Joint Logistic Support Force, from Jan 2006 to Mar 2023. The clinical characteristics of the patients are provided in Table 1. Notably, all participants were informed of the study via written consent and agreed to the publication of identifying details. The study protocol was approved by the Ethics Committee of the 989th Hospital of the PLA Joint Logistic Support Force review board.

### 2.2. Immunohistochemical Assay

Tumor samples were fixed in 4% paraformaldehyde solution with neutral phosphate-buffered solution (PBS; pH 7.2) and embedded in paraffin for sectioning (2–5 μm). Hematoxylin and eosin (H&E) staining was carried out for routine histopathological analysis. For immunohistochemical analyses [5], deparaffinized sections were heated under high pressure for antigen retrieval in 10 mM citrate buffer (pH 6.0). The sections were incubated with various dilutions of primary antibodies at 4 °C overnight (Table 2). After incubation with biotinylated secondary Abs at room temperature (RT) for 1 h, the results were visualized with DAB. A negative control was included by omitting the primary antibodies and substituting with corresponding normal isotype-matched immunoglobulin IgG. ASPP1 and iASPP positivity involves the cytoplasm, and ASPP2 protein positivity involves the cell nucleus. Cells were assessed as positive with the occurrence of yellow nuclei or brown particles. In addition, the staining intensity of the tumor cells was scored as negative (0 point), light yellow (1 point), deep yellow (2 points), or brown (3 points). And the scoring criteria for the percentage of positive cells was: 0 points for <1% positive cells, 1 point for 1–25%, 2 points for 26–50%, 3 points for 51–75%, and 4 points for >75%. The product of the staining intensity score and the percentage of positive cells are used as the final results, with ≥3 points considered positive. The results were interpreted by 2 senior pathologists independently.

### 2.3. Immunofluorescence Staining

To determine the precise location of ASPPs and identify the types of cells in LCS, we performed immunofluorescence analysis, as previously described [6]. Briefly, after deparaffinization, the sections were incubated with rabbit anti-Langerin mAb (1:200) and anti-iASPP pAbs (1:800) or anti-Langerin mAb (1:200) and anti-ASPP2 mAb (1:1000) at 4 °C overnight. After incubation with Alexa568-conjugated goat anti-mouse/rabbit IgG antibodies or fluorescein isothiocyanate (FITC)-conjugated mouse anti-goat IgG antibodies (Jackson ImmunoResearch, West Grove, PA, USA) for 1 h in a dark, moist chamber, the slides were mounted using fluorescence medium with 4,6-diamidino-2-phenylindole (DAPI) and observed under a fluorescence microscope. Fluorescent images were digitally merged. The results of immunofluorescence staining were analyzed using fluorescence microscopy (Zeiss Axioplan 2, Oberkochen, Germany).

### 2.4. Genetic Assay and Molecular Detection

To determine the tumor cell origin, prognosis, and screening for potential therapeutic molecular targets of LCS, including the BRAF V600E mutation, ROS-1 gene fusion and K-ras gene mutations detection were carried out. Analyses were conducted using amplification refractory mutation system (ARMS) technology in LCS, and one case of LCH was used as an internal control. At the same time, whole-exon sequencing was performed to explore the molecular mechanism of LC neoplasms. Briefly, DNA was extracted from 6 LCS samples (formalin-fixed and paraffin-embedded types) and confirmed by histopathologists. PCR products were purified using the DNA Fast Kit (Takara, Dalian, China) and sequenced directly with the Agilent SureSelect XT Human All Exon kit following the manufacturer’s instructions. Paired-end sequencing methods were used on the HiSeq2000 Genome Analyzer (Illumina) (Novo gene, Beijing, China), and sequencing libraries were constructed from 1 µg of DNA. Candidate variants were screened and analyzed using ESP6500, ExAC, the 1000 genomes project, and the Cancer Genome Atlas (TCGA) to obtain SIFT scores and Polyphen2 prediction.

## 3. Results

### 3.1. Expression of ASPPs Family Members in Langerhans Cell Neoplasms

**Figure 1:**
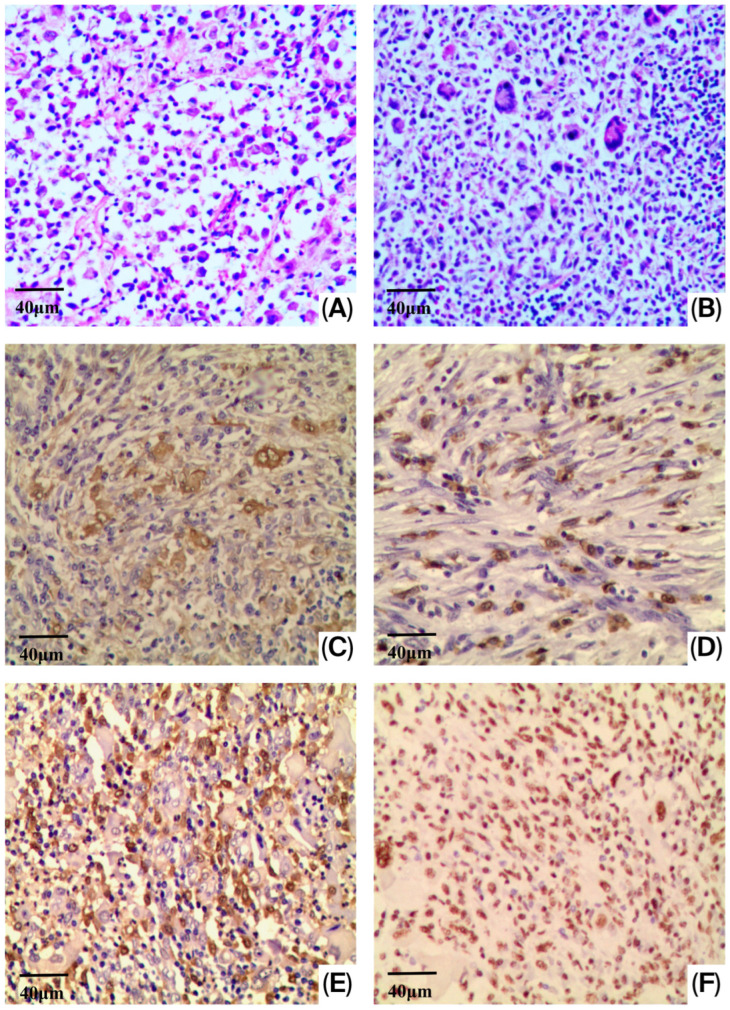
Histopathological and immunohistochemical analyses of LCS and LCH. Histopathological examination was performed by using hematoxylin-eosin staining in the tissue samples of LCH (**A**) (original magnification, ×200) and LCS (**B**) (original magnification, ×400) group. Immunohistochemical analyses was also performed by using CD1a (**C**) (original magnification, ×200), Langerin (**D**) (original magnification, ×200), WT-1 (**E**) (original magnification, ×200) and S-100 protein (**F**) (original magnification, ×200) in the tissue samples of LCS.

There were no significant differences in the tissue samples of LCH and LCS from conventional histopathological examination, except that LCS cells had more significant atypia. The tumor cells showed irregular shape with abundant and eosinophilic cytoplasm: large and irregular-shaped nuclei and prominent nucleoli. These cells displayed significantly malignant cytological features, and at the same time, the high mitotic rate (more than 10 mitoses per 10 high power fields) was observed (Figure 1A,B). In addition, consistent with a previous study, CD1a-, Langerin-, WT-1- and S-100 protein immunoreactions were observed in LCS (Figure 1C–F). We also detected the TP53 expression in the LCS and LCH; the results showed that the neoplastic LCs were negative; however, the IHC results cannot reliably distinguish wild-type from mutant TP53, and sequencing would be required for confirmation.

**Figure 2:**
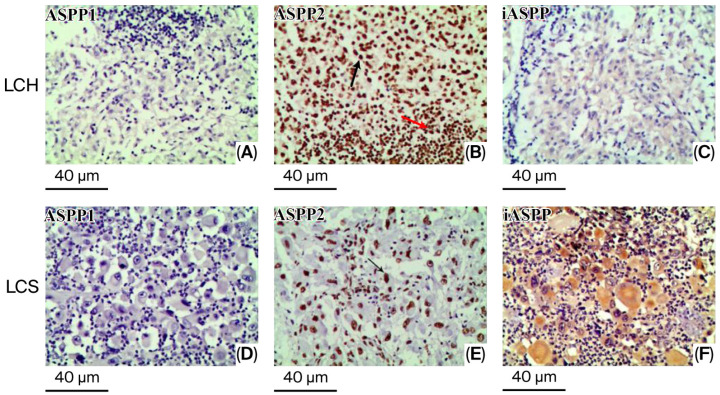
The expression of ASPP1, ASPP2, and iASPP in tissue samples of LCH and LCS was detected by immunohistochemical method. The LCH and LCS tissue samples were negative for ASPP1 (**A**,**D**) (original magnification, ×200). The expression of ASPP2 was observed on the aggregated tumor cell in LCS (**E**) (original magnification, ×200), but in LCH, tumor cells and histiocytic cells were both positive for ASPP2 (**B**) (original magnification, ×200). Black arrow heads indicate tumor cells and red arrows indicate histiocytic cells. iASPP immunoreaction was positive in the LCS tissue samples (**F**) (original magnification, ×200), and iASPP immunoreaction was negative in the LCH tissue sample (**C**) (original magnification, ×200).

Subsequently, no ASPP1 immunoreaction was observed in LCH or LCS (Figure 2A,D). In the case of LCH, ASPP2 was expressed not only in ovoid and elliptic cells, that is, proliferating Langerhans cells, but also in histiocytic cells, including neutrophils, plasma cells, eosinophils, and lymphocytes (Figure 2B). Moreover, in all samples of LCS (6/6), ASPP2 was observed in the nuclei of ovoid and elliptic neoplasm cells (Figure 2E). By contrast, a positive iASPP immunoreaction was observed in the cytoplasm of tumor cells in five cases of LCS (5/6) (Figure 2F), and no positive reaction was observed in LCH (Figure 2C). The intensities of ASPP2 and iASPP immunoreactivity were homogeneous in all samples examined.

**Figure 3:**
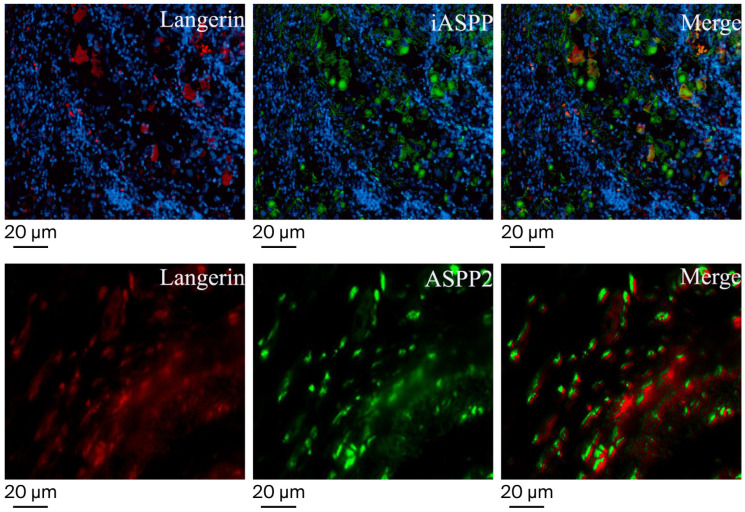
Determination of cell types expressing ASPP2 and iASPP in tissue samples of LCS. Triple-color immunofluorescence images are shown using anti-ASPP2, anti-iASPP, and anti-Langerin, as indicated at the top right of each panel. Signals were merged digitally in the right panel of each row (original magnification, ×400).

To determine the types of ASPP2- and iASPP-expressing cells present, we performed immunofluorescence staining using anti-Langerin, a marker for human Langerhans cells, and anti-ASPP2 or anti-iASPP antibodies. As shown in Figure 3, ASPP2-positive ovoid and elliptic neoplasm cells showed a positive signal for Langerin, suggesting that Langerhans cells also express ASPP2. Similarly, most iASPP-positive ovoid and elliptic tumor cells can be identified as Langerin-positive when signals of iASPP and Langerin merge. Taken together, these findings demonstrate that iASPP- and ASPP2-expressing cells can be identified as Langerin-positive cells in LCS.

### 3.2. Molecular Genetic Detection

**Figure 4:**
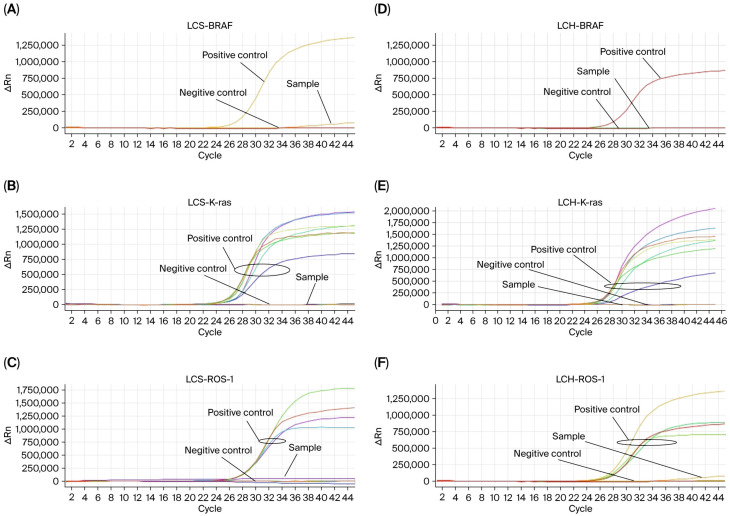
The results of BRAF V600E, ROS-1 and K-ras gene detection in LCH and LCS tissue samples. BRAF V600E in LCS (**A**); K-ras in LCS (**B**); ROS-1 in LCS (**C**); BRAF V600E in LCH (**D**); K-ras in LCH (**E**); ROS-1 in LCH (**F**).

Six cases of LCS and one case of LCH were detected for the BRAF V600E mutation, ROS-1 rearrangement, and K-ras gene mutation. And no mutations or gene fusions were detected in these samples (Figure 4).

### 3.3. WES Results of LCS Samples

**Figure 5:**
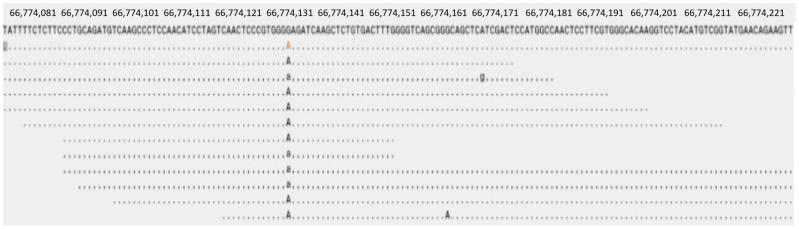
The results of NGS detection in LCS tissue samples, MAP2K1 gene c.607G > A. The red “A” represents the mutated base.

We performed WES in combination with target sequence validation on six LCS samples. Purified DNA from six LCS samples and peripheral normal tissue were analyzed using WES. The mean sequencing depths in the tumor and normal tissues were 100×. We identified a mutation in four LCSs; nonsynonymous mutations were found in 607 bp of MAP2K1. This resulted in a change from base G to base A, and a corresponding change in amino acids from E to K at site 203. The frequency of MAP2K1 variants c.607G > A was 6.17% in LCS (Figure 5). After analyzing the results again, we identified that MAP2K1 is a somatic driver mutation for LCS. Other common changes in genes are outlined in Supplementary Table S1 and Supplementary Figure S1.

## 4. Discussion

LCS is a rare malignant tumor that originates from LC with markedly malignant features. At present, approximately 58 cases of LCS have been reported in English and Chinese studies [7]. The total survival time of LCS ranges from 0.5 to 108 months, and the average survival time is 23.7 months. Arber, DA reported that the mortality rate of LCS is 50% [8], and 66.7% of patients die in the first 18 months, even though they are administered with systemic and/or local therapy [9,10]. Initially, it was believed that most cases of LCS involved a single organ or tissue, and only a small number of cases involved multiple organs [11]. However, an increasing number of studies have confirmed that a single site or organ is only identified in a minority of cases. Single organs include skin [12,13], bone [14,15], lymph nodes [16], breast [17], and lungs [13,18]. The majority of LCS cases involve multiple organs or sites through invasion and metastasis [10,19]. This further indicates that LCS is a highly aggressive and malignant tumor.

A few studies have been conducted on the molecular pathway and differential diagnosis between LCH and LCS [20,21,22]. Murakami I et al. reported that the viral load of Merkel cell polyomavirus was higher in LCS than LCH using multiplex quantitative PCR and immunohistochemical methods [20]. Previous research also indicated that LCs were positive for cancer-associated B7 molecules, such as B7-H1, B7-H3, B7-DC, Z39Ig, and B7, which may be potential biomarkers to identify LCS [21]. Another study showed that WT1 and CD44 are potential biomarkers for differentiating between LCH and LCS [22]. Nonetheless, the molecular mechanism, diagnostic difficulty, and specificity of LCS are still challenges in clinical practice. It is essential to study its molecular pathway and search for more specific biomarkers or key checkpoints for the differential diagnosis and treatment of LCS.

ASPP is a p53 protein downstream regulation molecule that was discovered by Samuels-Lev et al. in 2001 [23]. ASPP genes regulate cell growth, apoptosis, and differentiation in various neoplasms. The ASPP family consists of ASPP1, ASPP2, and iASPP. The function of ASPP1/ASPP2 is to combine with p53 to regulate the bioactivity of p53 proteins and promote cell apoptosis. In contrast, iASPP has opposite bioactivities and functions to oncogenes. Although the expression and function of ASPP family members in multiple neoplasms have been widely examined, their expression and distribution in LCS have not yet been studied.

In the present study, a positive ASPP1 immunoreaction was not observed in either LCH or LCS, which may suggest that the role of ASPP1 in the LC neoplasm is insignificant. An ASPP2-positive immunoreaction was observed in ovoid and elliptic tumor cells, but not in histiocytic cells, in six cases of LCS. By contrast, ASPP2 was expressed not only in proliferating LCs but also in histiocytic cells in LCH. These results indicated that the expression pattern of ASPP2 helps in the diagnosis of LCS. Moreover, Langerin+ tumor cells, not histiocytic cells, expressed ASPP2. Thus, our results show that ASPP2 may be involved in the development of LCS. Therefore, an ASPP2 expression pattern could serve as a potential molecular marker for the differential diagnosis of LCS. In this study, our results also indicate that iASPP is expressed in the cytoplasm of neoplastic LCs, not in proliferating LCs. This further suggests that ASPP2 and iASPP expression patterns may be involved in the development of LCS and could be used as promising markers for differentiation between LCS and LCH.

Having clarified the differential diagnostic issues between LCH and LCS by immunohistochemical methods, it is also necessary to study the molecular mechanisms of the malignant transformation of LCs to gain a deeper understanding of the pathogenesis of LCS. At the same time, the sequencing results may be potential markers for differentiating between LCH and LCS. Firstly, we detected the K-ras gene, BRAF V600E, and ROS-1 in LCS and LCH; the results found no evidence of gene mutations or rearrangements. While a previous study reported that BRAF mutations were detected in more than half of LCH cases [24] and no LCS patients [25], a recent study detected BRAF V600E mutations in cases of LCS [26]. From our results, recurrent chromosomal abnormalities were rare in LC neoplasms. Secondly, we performed the NGS, and the results showed that many genes remained normal in LCS except MAP2K1. MAP2K1 mutations were detected in four cases of LCS, encoding the negative regulatory domain of the MEK1 protein. However, the mutations in the MAP2K1 gene c.607G > A in patients with LCS caused changes in amino acids from glutamic (E) to lysine (K) at site 203. MAP2K1 (MEK1) mutations are found in approximately 25% of patients with LCH according to published papers [27,28]. In patients with LCH, MAP2K1 is an activating mutation that is mutually exclusive to BRAF V600E mutations according to the literature. At the same time, studies on the treatment of the MAP2K1 gene using trametinib have been reported in several cancer types [29,30], including histiocytosis [31,32]. However, the mutations of the MAP2K1 gene in patients with LCH resulted in a p.L98K104 > Q deletion in the published paper [33]; otherwise, in LCS, c.607G > A resulted in changes in amino acids from E to K at site 203 in our study. This is the first report of the MAP2K1 gene c.607G > A mutation in LCS. Tashakori reported no BRAF V600E or MAP2K1 mutations in LCS [34]; another paper showed that a MAP2K1 mutation was detected in a case of multi-organ LCS [35]. A study also suggests MAPK/PI3K signaling pathway is a potential dysregulation in part of LCS [36]. In addition, the BRAF V600E gene mutation was negative in six cases of LCS, which was different from LCH [37]. The c.607_E203 > K mutation of MAP2K1 in LCS has not been previously reported in the literature.

This study address molecular changes in LCS based on the expression of ASPP family members and MAP2K1 gene. The result indicates that expression pattern of ASPP2 and iASPP in LCs contributes to diagnosis of LCS. The MAP2K1 gene c.607G > A was identified as the promising marker in LCS. In summary, immunohistochemical detection of ASPP2 and iASPP expression, in conjunction with other biomarkers, can further enhance the promising diagnosis and therapy for LCS. The study also has limitations, mainly due to the small number of cases, with only 6 cases of LCS, the method of evaluation is also simple, and the cross-validation is not adopted. In the next stage of research, more cases will be included for molecular detection, the spatial omics analysis will be done, and a more expansive validation will need to be performed to ensure the objectivity of the conclusions.

## 5. Conclusions

This study implies that ASPP2 and iASPP are potential markers that can be used to distinguish LCS from LCH. The MAP2K1 gene c.607G > A leads to changes in amino acids at the 203 site from E to K and is also a promising marker of LCS. Thus, combined analysis of ASPP expression pattern, molecular detection and other reported biomarkers should be carried out to more accurately differentiate between LCS and LCH.

## Figures and Tables

**Table 1:** Clinical characteristics of patients with LCS and LCH.

Case	Gender	Age (Years)	Involving Site	Diagnostic Method	Diagnosis	Therapy	Outcome
1	M	41	Soft tissue at anterior iliac spine and bilateral groin	Pathology, Computed tomography, MRI	LCS	Surgery, RT, CT	DOD, 12 months
2	M	37	Soft tissue at waist	Pathology, MRI	LCS	Surgery, CT	DOD, 5 months
3	M	57	Right lower extremity	Biopsy + pathology	LCS	Surgery, CT	DOD, 8 months
4	FM	34	Lymph node	Biopsy + pathology	LCS	RT, CT	DOD, 2 months
5	M	61	Anal canal	Biopsy + pathology	LCS	Surgery, CT	Survived, 25 months
6	FM	65	Lymph node	Pathology	LCS	Surgery, CT	Survived, 10 months
7	M	12	skull	Biopsy + pathology	LCH	Follow-up	Survived, 152 months
8	FM	5	skull	Biopsy + pathology	LCH	Follow-up	Loss to follow-up
9	FM	26	ribs	Surgical excision + pathology	LCH	Follow-up	Survived, 12 months
10	FM	46	Tympanic sinus	Biopsy + pathology	LCH	Follow-up	Survived, 39 months
11	M	5	skull	Surgical excision + pathology	LCH	Follow-up	Survived, 6 months
12	M	51	ribs	Biopsy + pathology	LCH	Follow-up	Survived, 3 months

M, male; FM, female; MRI, magnetic resonance imaging; surgery, surgical excision; RT, radiotherapy; CT, chemical therapy; DOD, died of disease; LCS, Langerhans cell sarcoma; LCH, Langerhans cell histiocytosis.

**Table 2:** Primary antibodies used in this study and their source.

Primary Ab	Dilution	Clone	Source
ASPP1	1:300	Mouse anti-human ASPP1 monoclonal antibody (LX011)	Santa Cruze
ASPP2	1:1000	Rabbit anti-human ASPP2 monoclonal antibody	Abcam
iASPP	1:800	Rabbit anti-human iASPP multi-clonal antibody	Abcam
CD20	1:200	Monoclonal mouse IgG	DAKO
LCA	1:50	Monoclonal mouse IgG	DAKO
CD3	1:50	Monoclonal mouse IgG (F7.2.38)	DAKO
MPO	1:50	Monoclonal rabbit IgG (ab208670)	Abcam
CD68	1:50	Monoclonal mouse IgG (3F103)	Santa Cruze
CD56	1:50	Monoclonal mouse IgG (ERIC-1)	Millipore
Langerin	1:200	Monoclonal mouse IgG (12D6)	Abcam
CD1a	1:200	Monoclonal mouse IgG (7A7)	Abcam
S-100 protein	1:800	Monoclonal mouse IgG (8B10)	Abcam
CD44	1:25	Monoclonal mouse IgG	DAKO
WT-1	1:50	Monoclonal mouse IgG	DAKO
TP53	1:100	Monoclonal mouse IgG	Maxin Biotech

## Data Availability

All data are available upon request to the corresponding author.

## Supplementary Materials

The following supporting information can be downloaded at: https://www.mdpi.com/article/10.3390/diagnostics16101418/s1, Figure S1: The results of WES detection in LCS tissue samples; Table S1: Gene status in LCS detected by WES.

## Abbreviations

The following abbreviations are used in this manuscript:

ASPP	apoptosis-stimulating protein of p53
LCS	Langerhans cell sarcoma
iASPP	inhibitory member of the ASPP family
LCH	Langerhans cell histiocytosis
ARMS	amplification refractory mutation system
MAP2K1	mitogen-activated protein kinase kinase1
WES	whole exon sequencing
Abs	antibodies
Q-PCR	quantitative PCR
