# Supplementary material for: Differential Expression of Apoptosis-Stimulating Proteins of p53 (ASPPs) Between Langerhans Cell Histiocytosis and Langerhans Cell Sarcoma
Source: Diagnostics (Basel). 2026 May 7;16(10):1418. doi: 10.3390/diagnostics16101418 (PMC13205305; doi:10.3390/diagnostics16101418)
Supplement: Supplementary file 1 [file diagnostics-16-01418-s001.zip › diagnostics-4199213-supplementary.pdf]

Supplementary Table S1. Gene status in LCS detected by WES.

Gene	Site	Result
EGFR	(Exon18) G719<br>(Exon19) del<br>(Exon21) L858R<br>(Exon21) L861Q<br>(Exon20) T790M<br>(Exon20) ins<br>(Exon20) C797S<br>Amp	Non-mutation.
KRAS	(Exon2) G12/13,<br>(Exon3) A59/Q61<br>(Exon4) K117/A146	Non-mutation.
MET	Amp<br>Exon14 splicing	Negative.
ALK	Fusion<br>(Exon23) p.L1196<br>(Exon23) p.S1206<br>(Exon25) p.G1269<br>(Exon22) p.T1151_L1152ins	Negative.
ERBB2	Amp<br>(Exon20) ins	Negative.
CD274	Amp	Negative.
ROS1	Fusion, G2032	Negative.
DDR2	(Exon18) S768R	Negative.
BRAF	(Exon15) V600<br>(Exon15)Y472C<br>(Exon11)G466V	Negative.
NRAS	(Exon2) G12/13<br>(Exon3) A59/Q61<br>(Exon4) A146	Negative.
RET	Fusion<br>(Exon16) M918T	Negative.
PIK3CA	(Exon9)E542<br>(Exon9) E545<br>(Exon20) H1047	Negative.
PTEN	CNV Loss	Negative.
TSC1	(Exon15) p.E636fs	Negative.
BCR-ABL1	Fusion	Negative.
BRCA1/ BRCA2	Deleterious Mutation	Negative.
CDK4	Amp	Negative.
FGFR1	Amp	Negative.

FGFR2	Amp	Negative.
MMR(MLH1、MSH2、MSH6、PMS2)	Deleterious Mutation	Negative.
Kit	(Exon9)<br>(Exon11)<br>(Exon17) D816V<br>(Exon 13)	Negative.
CDK6	Amp	Negative.
SMO	(Exon8) D473H	Negative.
PDGFRA	(Exon12) V561<br>(Exon14) N659<br>(Exon18) D842V	Negative.

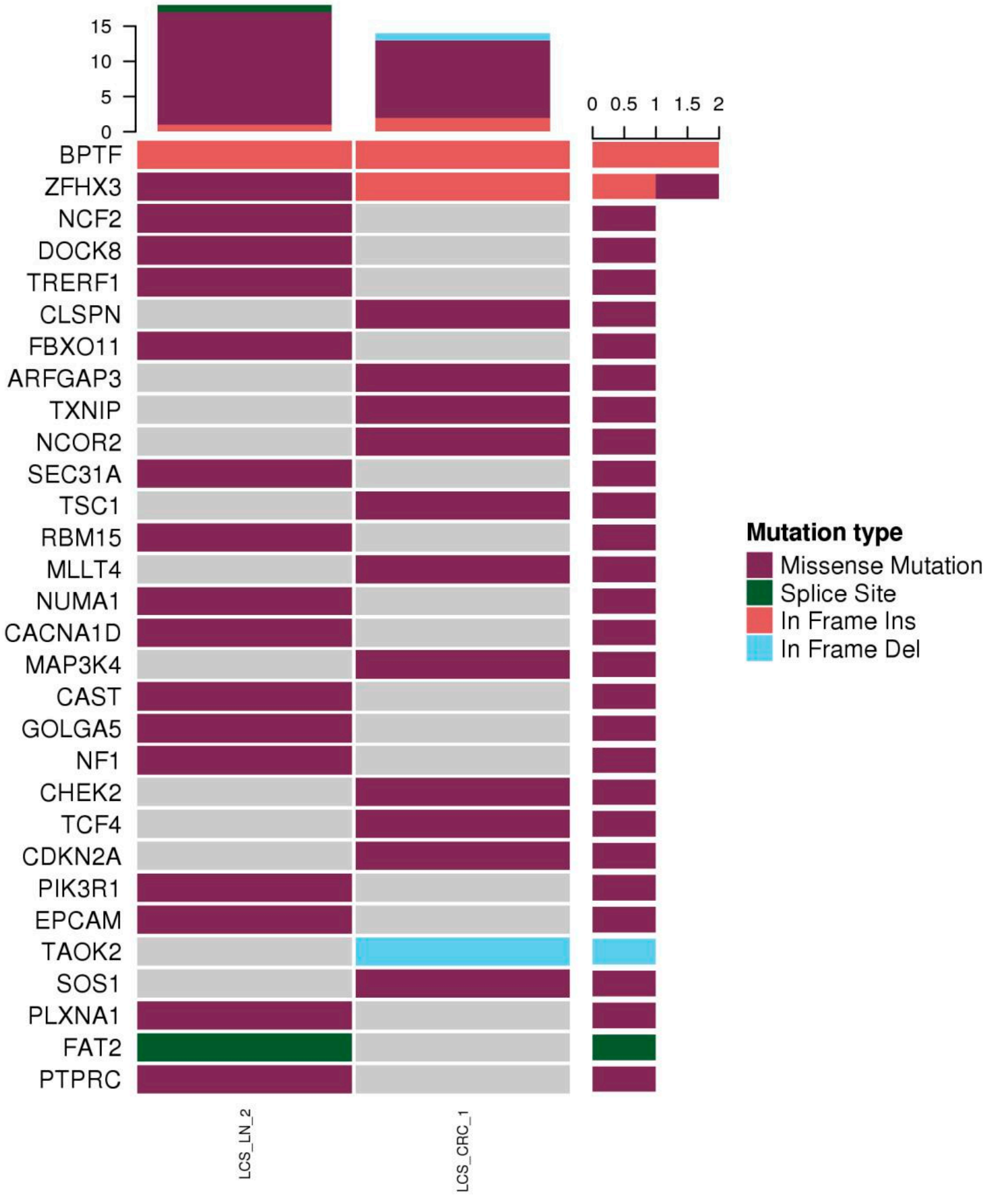

Supplementary Figure S1. The results of WES detection in LCS tissue samples.
